# Simultaneous Determination of Nitroimidazoles and Quinolones in Honey by Modified QuEChERS and LC-MS/MS Analysis

**DOI:** 10.1155/2018/4271385

**Published:** 2018-01-01

**Authors:** Haiyan Lei, Jianbo Guo, Zhuo Lv, Xiaohong Zhu, Xiaofeng Xue, Liming Wu, Wei Cao

**Affiliations:** ^1^Department of Food Science and Engineering, School of Chemical Engineering, Northwest University, Xi'an, Shaanxi 710069, China; ^2^Shaanxi Institute for Food and Drug Control, Xi'an, Shaanxi 710069, China; ^3^Institute of Apiculture Research, Chinese Academy of Agricultural Sciences, Beijing 100093, China

## Abstract

This study reports an analytical method for the determination of nitroimidazole and quinolones in honey using liquid chromatography-tandem mass spectrometry (LC-MS/MS). A modified QuEChERS methodology was used to extract the analytes and determine veterinary drugs in honey by LC-MS/MS. The linear regression was excellent at the concentration levels of 1–100 ng/mL in the solution standard curve and the matrix standard curve. The recovery rates of nitroimidazole and quinolones were 4.4% to 59.1% and 9.8% to 46.2% with relative standard deviations (RSDs) below 5.2% and the recovery rates of nitroimidazole and quinolones by the matrix standard curve ranged from 82.0% to 117.8% and 79% to 115.9% with relative standard deviations (RSDs) lower than 6.3% in acacia and jujube honey. The acacia and jujube honeys have stronger matrix inhibition effect to nitroimidazole and quinolones residue; the matrix inhibition effect of jujube honey is stronger than acacia honey. The matrix standard curve can calibrate matrix effect effectively. In this study, the detection method of antibiotics in honey can be applied to the actual sample. The results demonstrated that the modified QuEChERS method combined with LC-MS/MS is a rapid, high, sensitive method for the analysis of nitroimidazoles and quinolones residues in honey.

## 1. Introduction

**Figure 1:**
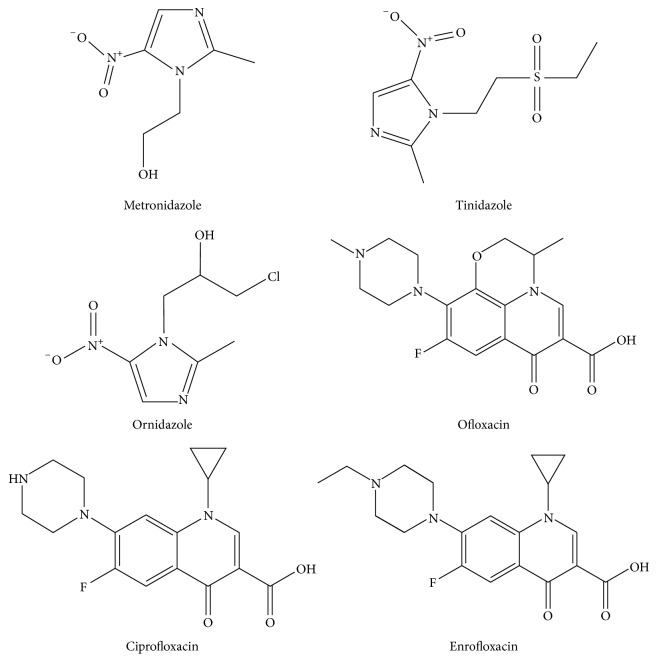
Chemical structures of six nitroimidazoles and quinolones.

Nitroimidazoles and quinolones (Figure 1) are a group of antibacterial compounds that have been widely used in medical domain. There are many antibiotics left in honey because of the illegal addition of beekeepers [1–4], which directly threatens the health and safety of consumers. Nosemosis of bees is one of the protozoa infections in adult honeybee, which is very destructive to honeybee colonies and is an infectious disease caused by* Cryptosporidium parvum*. Nitroimidazoles, for example, metronidazole, can be used to prevent and treat nosemosis of bees. Quinolones, for instance, ofloxacin, can potentially be used to prevent and treat honeybees piroplasmosis. However, the misuse and illegal use of nitroimidazoles drugs may cause potential hazards of cell mutagenicity and carcinogenic radionuclide, quinolones drugs can lead to the reaction of certain degree and hepatotoxicity or even death [5, 6]. Therefore, nitroimidazoles and quinolones have been banned in honey. Hence, the control of nitroimidazoles and quinolones is highly significant for the agricultural environment and food industry.

High-performance liquid chromatography (HPLC) with diode array detector (DAD) [7], liquid chromatography with fluorescence detection (LC-FD) [8], liquid chromatographic-mass spectrometric (LC-MS) [9, 10], and liquid chromatography-tandem mass spectrometry (LC-MS/MS) have been used to analyze nitroimidazoles and quinolones in food industry (e.g., milk powder, bovine milk, butter, fish tissue, eggs, chicken meat, pig plasma, bovine meat, swine tissues, honey, feed hair, and water) [11–19]. LC-MS/MS is one of the most promising techniques for the analysis of antibiotics in foodstuff because of its sensitivity and accuracy. Sample preparation generally use QuEChERS approach, SPE clean-up or liquid-liquid extraction. Compared with SPE clean-up and liquid-liquid extraction, QuEChERS approach has almost the same purifying effect, but it requires minimum operational steps and solvent and has higher accuracy and wider application [16, 20–24]. It was initially developed for the analysis of pesticide residues in fruits and vegetables and was extended to the analysis of veterinary drugs and environmental pollutants residues [25].

**Figure 2:**
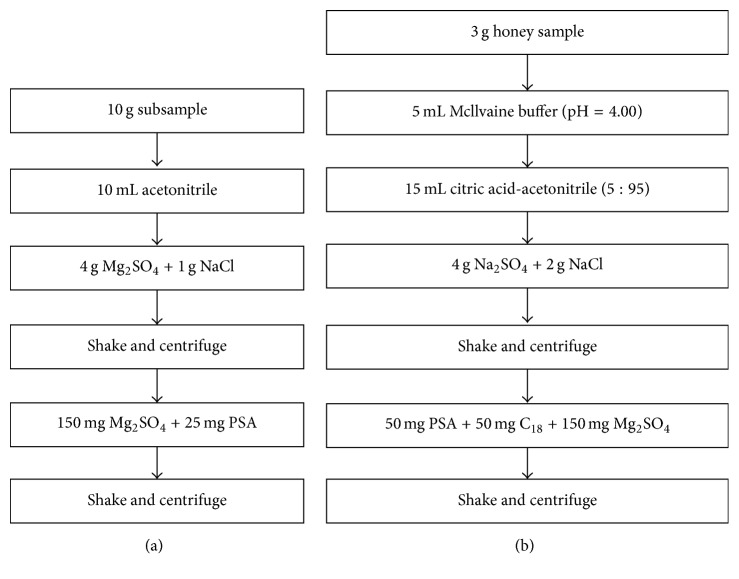
Original QuEChERS methodology (a) and modified QuEChERS methodology (b).

In this study, we developed a multiresidue test method based on the application of LC-MS/MS combined with modified QuEChERS sample preparation methodology for rapid determination of nitroimidazoles and quinolones residues. The improvement of the method is shown in Figure 2.

The innovation of this method is to eliminate the matrix effect in honey via matrix effect standard curve; the matrix effect of acacia and jujube honey has stronger inhibitory action to nitroimidazoles and quinolones residue with the matrix inhibition effect of jujube honey being stronger than acacia honey. The matrix standard curve can effectively correct the matrix effect in honey. This method is more accurate than the previous published method.

## 2. Experimental

### 2.1. Materials and Reagents

Acacia and jujube honey samples were purchased from consumer stores and provided by beekeepers. The samples were stored at ambient temperature (25°C) before analysis.

Analytical standard substances, including metronidazole (purity = 100.0%) CAS: 443-48-1, batch lot: 100191-201507; ornidazole (purity = 100.0%) CAS: 16773-42-5, batch lot: 100608-201102; tinidazole (purity > 99.9%) CAS: 19387-91-8, batch lot: 100336-200703; ofloxacin (purity > 99.5%) CAS: 82419-36-1, batch lot: 130454-201206; ciprofloxacin (purity > 99.5%) CAS: 85721-33-1, batch lot: 130451-201203, were obtained from Institute of Pharmaceutical and Biological Products (Beijing, China); enrofloxacin (purity > 99%) CAS: 93106-60-6, batch lot: 107071, was purchased from Dr. Ehrenstorfer GmbH (Augsburg, Germany).

These reagents of sodium chloride, sodium hydroxide, anhydrous sodium sulfate, anhydrous magnesium sulfate, citric acid, disodium hydrogen phosphate, and glacial acetic acid are of analytical purity (Sinopharm, Beijing, China).

Ammonium formate (Fluka, Tianjin, China), formic acid (Fluka, Tianjin, China), acetonitrile (Fisher, Fairlawn, USA), and methanol (Tedia, Fairfield, USA) are of HPLC grade. PSA and C_18_ (40 *μ*m) are of also analytical purity (Agela, Beijing, China). A Milli-Q ultrapure water system (Millipore, Bedford, MA, USA) was used to obtain the HPLC-grade water.

Anhydrous sodium sulfate was baked at 600°C for 3 h and moved the sealed container for conservation.

Mcilvaine buffer (pH = 4.00) was prepared by dissolving 19.2 g disodium hydrogen phosphate and 8.9 g citric acid in 1.625 L of Milli-Q water and the pH was adjusted with 4 mol·L^−1^ sodium hydroxide solution.

### 2.2. Standard Solutions

The individual stock standard solutions of metronidazole, ornidazole, tinidazole, ofloxacin, ciprofloxacin, and enrofloxacin were prepared in methanol at the concentration of 1 mg/mL. The mixed working standard solutions (1 *μ*g/mL) were prepared by diluting stock solutions with methanol. All standard solutions were stored at −20°C in dark bottles.

Preparation of standard solutions: mixture working solutions (1 *μ*g/mL) were diluted with methanol/water (50/50, v/v) at the concentration of 1, 5, 10, 20, 40, and 100 ng/mL.

The matrix-matched working standard solutions: 3.0 g homogenized negative acacia and jujube honey samples were weighed and placed into 50 mL polypropylene centrifuge tube, respectively; then various amounts mixture working solutions were added.

### 2.3. Sample Preparation

The modified QuEChERS methodology was used. An aliquot of 3.0 g homogenized samples was weighed and placed into 50 mL polypropylene centrifuge tube, and 5 mL Mcilvaine buffer (pH = 4.00) was added. The mixture was shaken in a vortex mixer for 30 s; then 15 mL citric acid- acetonitrile (5 : 95) was added. Subsequently, 2.0 g sodium chloride and 4.0 g anhydrous sodium sulfate were added to this mixture and vigorously shaken in a vortex for 2 min; afterwards, the tube was centrifuged at 10000 rpm for 10 min. Next, 10 mL supernatant solution was transferred into a 15 mL centrifuge tube and evaporated to 2 mL under nitrogen at 40°C. Then 50 mg PSA, 50 mg C_18_, and 100 mg anhydrous Mg_2_SO_4_ were added to the tube in a vortex for 2 min and then centrifuged at 10000 rpm for 10 min. Next, 1 mL supernatant solution was transferred into a 15 mL centrifuge tube and evaporated to dryness under nitrogen at 40°C. The residue was reconstituted in 1 mL methanol/water (50/50, v/v) and filtered through a 0.45 *μ*m filter before LC-MS/MS analysis.

### 2.4. LC-MS/MS Conditions

#### 2.4.1. LC Conditions

Chromatographic analyses were performed by Waters 2695 series HPLC System (Milford, MA, USA); chromatographic separation was achieved by Waters XTerra RP18 (2.1 mm × 150 mm, 5 *μ*m) analytical column. The injection volume was 10 *μ*L, and the temperature of the column was maintained at 35°C. The mobile phases were acetonitrile (mobile phase A) and 10 mM ammonium formate + 0.5% formic acid in Milli-Q water (mobile phase B) at a flow rate of 0.3 ml/min. The total chromatographic runtime was 15 min. Mobile phase gradient flow program was shown in Table 1.

#### 2.4.2. MS/MS Conditions

Waters Quattro Micro API triple quadruple tandem MS coupled to electrospray ionization (ESI) interface and Waters Jet Stream Ion Focusing (Waters, USA) was used for mass analysis and quantification of target analytes. The MS was operated in the positive ion mode and utilized multiple reaction monitoring (MRM). The tuning parameters were optimized for the target analytes: the gas temperature was set at 350°C with a flow rate of 800 L/h and the gas was high purity nitrogen. Capillary voltage was 2.5 kV, ion source temperature was 120°C, cone gas flow was 50 L/h, and the gas was high purity nitrogen. The system operation, data acquisition, and analysis are controlled and processed by the MassHunter software.

## 3. Results and Discussion

### 3.1. Optimization of Mass Spectrometry Conditions

Both positive and negative ionization modes in ESI were used to evaluate the signal responses of target analytes [26]. After evaluation, the target compounds (metronidazole, tinidazole, ornidazole, ofloxacin, ciprofloxacin, and enrofloxacin) were analysed in positive ESI mode ([M + H]+) due to higher response. The size of collision energy and cone voltage influence sensitivity and fragmentation [22]. In the full-scan mass analysis, the parent ion is obtained; then the optimum cone voltage was optimized according to higher response. The fragmentation ion of every target compound was obtained via product scan in optimum cone voltage. The fragment ion of metronidazole is 128.0 m/z and 81.9 m/z, the fragment ion of tinidazole is 128.0 m/z and 92.8 m/z, the fragment ion of ornidazole is 127.9 m/z and 81.9 m/z, the fragment ion of ofloxacin is 261.0 m/z and 221.0 m/z, the fragment ion of metronidazole is 314.0 m/z and 231 m/z, and the fragment ion of metronidazole is 245.0 m/z and 203 m/z. The collision energies were optimized for each individual analyte to give the best response. The most intense and stable fragmentation ions were selected for quantification ion, and the second most abundant ions were used for qualification ion [27]. Eventually, the best collision energies of quantification ion and qualification ion were optimized by the maximum intensity response. All the MS/MS parameters are presented in Table 2.

### 3.2. Matrix Effects

**Figure 3:**
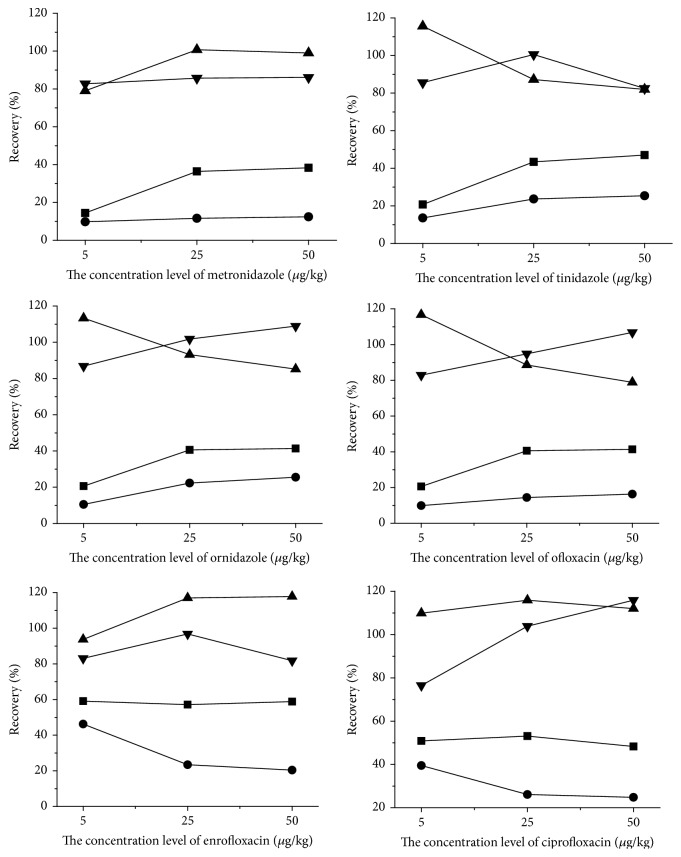
Absolute and relative recovery of target compounds. ■, ●: the recovery rates of six kinds of drugs by the solution standard curve in acacia and jujube honey, respectively; ▼, ▲: the recovery rates of six kinds of drugs by the matrix standard curve in acacia and jujube honey, respectively.

Matrix is components of exclusive tested matter, which has a significant interference for linearity, accuracy, precision, limits of detection, and quantification. The interference was said to be matrix effect. Recently, they have been discussed in several review articles [27, 28]. Matrix effects (MEs) are a major problem affecting the quantitative accuracy of liquid chromatography-electrospray ionization mass spectrometry (LC-ESI-MS) when analyzing complicated samples [1]. The influence of sample substrate on target compounds determination is derived from endogenous components of sample, which are organic and inorganic components and exist in extracting solution after sample preparation; the components included ion particle composition (electrolytes and salts), strong polar compounds (phenols and pigment), and organic components (sugars, amines, carbamide, lipoid, congener, and metabolism of target object); when these substances and target compounds fly into the ion source at the same time after honey samples preparation, they will affect the ionization process of target compounds [29]. In the study, the matrix effects in these samples were evaluated by the spiked honey samples at 5, 25, and 50 *μ*g/kg. According to Figure 3, compared with matrix-matched calibration curve, the recovery of the neat standard calibration curve was lower, and ME indicated matrix suppression. ME phenomena were obvious in honey sample, which may be impressed by sugars, phenols, pigment, protein, and flavonoids. Moreover, jujube honey contain high contents of pigment and flavonoids that make it dark; therefore, recovery of jujube honey is lower than acacia honey and the matrix effect of jujube honey was stronger than acacia honey. Hence, matrix-matched standard curve has been used to overcome the matrix effect and signal irreproducibility, matrix interference, and loss of recovery. Figure 3 showed that the matrix-matched working standard curves for each compound can meet detection requirements and effectively correct matrix effects in acacia and jujube honey.

### 3.3. Linearity

A serial dilution of the standard mixture was prepared (1–100 ng/mL) and analyzed using the optimized assay conditions. And the correlation coefficients (*R* ^2^) ranged within 0.994–0.999 using this method. The negative acacia and jujube honeys were extracted and tested according to Sections 2.3 and 2.4. The matrix-matched calibration curve was constructed by determining the peak area of metronidazole, ornidazole, tinidazole, ofloxacin, ciprofloxacin, and enrofloxacin at six concentration levels in the range of 10–100 ng/mL curve in acacia and jujube honey and the correlation coefficients (*R* ^2^) ranged within 0.990–0.999 and 0.992–0.996, respectively. In general, the linearity of the matrix-matched working standard curves and the solution standard curve was excellent. The data were treated by using the Waters QuanLynx module of the MassLynx software. The result is shown in Table 3.

### 3.4. Limit of Detection and Quantification

The limits of detection (LOD) and limits of quantification (LOQ) were determined (*n* = 3) via the signal-to-noise ratio (SNR) of the analyte; LOD and LOQ were estimated as 3 × S/N and 10 × S/N, respectively. The LODs of the target compounds were achieved in the range of 0.64–1.41 *μ*g/kg and 0.83–1.58 *μ*g/kg for acacia and jujube honey, respectively. The LOQs of the target compounds were achieved in the range of 2.13–4.70 *μ*g/kg and 2.77–5.27 *μ*g/kg for acacia and jujube honey, respectively. The result is shown in Table 4.

### 3.5. Recovery and Precision

**Figure 4:**
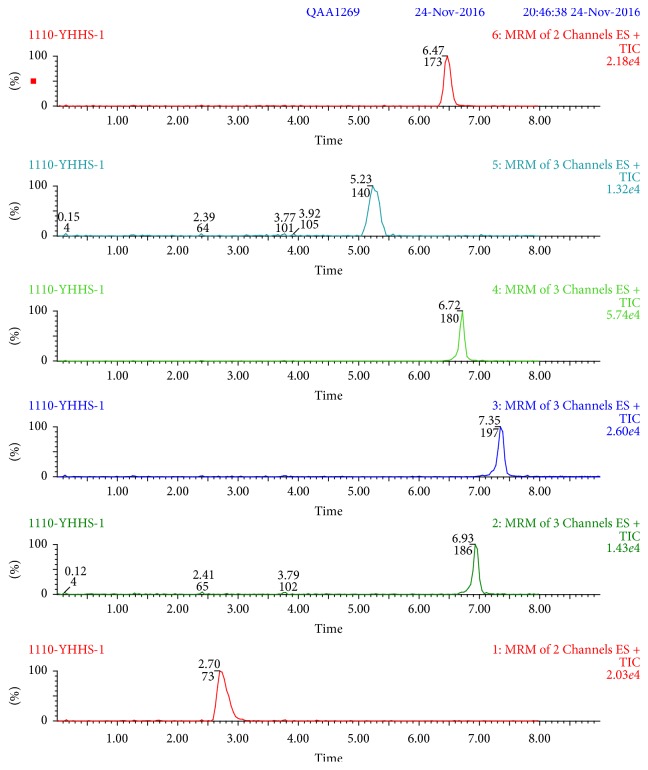
Typical chromatograms of MRM transitions for metronidazole, tinidazole, ornidazole, ofloxacin, ciprofloxacin, and enrofloxacin at the concentration of 25 *μ*g/kg in acacia honey.

**Figure 5:**
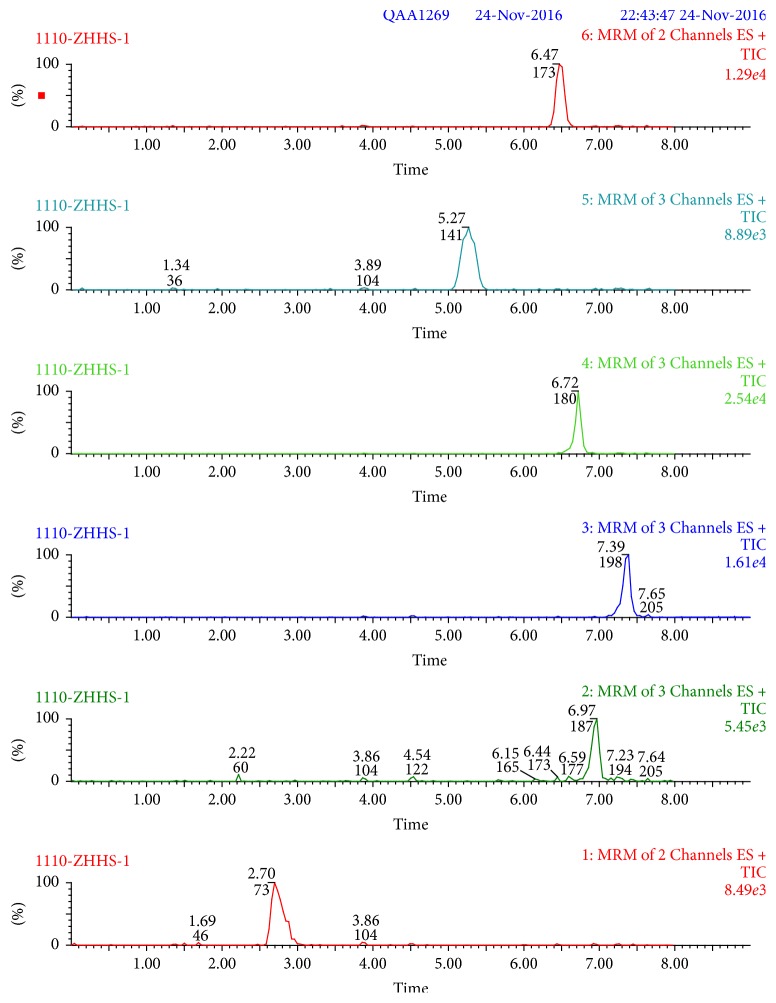
Typical chromatograms of MRM transitions for metronidazole, tinidazole, ornidazole, ofloxacin, ciprofloxacin, and enrofloxacin at the concentration of 25 *μ*g/kg in jujube honey.

Recovery and precision were determined at three concentration levels (5, 25, and 50 *μ*g/kg) for acacia and jujube honey. Recoveries of the analytes ranged within 81.9%–116.8% and 81.0%–115.9% in acacia and jujube honey, respectively. In detail, recoveries range within 81.0% −100.7% (metronidazole), 82.5%–115.6% (tinidazole), 85.2%–113.3% (ornidazole), 81.9%–116.7% (ofloxacin), 81.8%–116.8% (ciprofloxacin), and 86.5%–115.9% (enrofloxacin). Recoveries for target compounds were higher than 80% and RSDs were lower than 6.3% in Table 5. These are highly acceptable values for the honey samples. Typical chromatograms of MRM transitions about six drugs are shown in Figures 4 and 5 at the concentration of 25 *μ*g/kg in acacia and jujube honey.

### 3.6. Application to Real Samples

The proposed modified QuEChERS method with LC-MS/MS was applied to 46 actual honey samples from honey producers and cooperatives located in the cities of Shaanxi, Hebei, Gansu, Chongqing, Hubei, and Shanxi in China. The samples were extracted and analysed according to the protocols described in Sections 2.3 and 2.4. Quantifications of metronidazole, tinidazole, ornidazole, ofloxacin, ciprofloxacin, and enrofloxacin were performed using the external standard. The results are shown in Table 6.

## 4. Conclusion

A simple and rapid method of modified QuEChERS combined with liquid chromatography-tandem mass spectrometry for the determination of multiresidue in honey samples was established. The method was fast, efficient, reliable and could be used in the monitoring of antibiotic in honey, which is fit for the purpose and satisfactory in terms of selectivity, recovery, and accuracy. Therefore, it has been successfully applied to the determination of nitroimidazoles and quinolones residues. Matrix standard calibration curves were successfully employed in correction for matrix effect. It can be seen that some honeys have the residues of nitroimidazole and quinolones in Table 6. Hence, the control of antibiotics in food is highly significant for the agricultural environment and food industry.

## Figures and Tables

**Table 1:** Mobile phase gradient flow program.

Time/min	A/%	B/%
0.00	5	95
3.00	20	80
8.00	25	75
8.10	50	50
10.00	50	50
10.10	5	95
15.00	5	95

A: acetonitrile; B: 10 mM ammonium formate + 0.5% formic acid in Milli-Q water.

**Table 2:** Retention time and MS/MS conditions for the target compounds.

Name	Qualification ion (m/z)	Quantification ion (m/z)	Cone voltage (V)	Collision energy (eV)	ion ratio	Retention time (min)
Metronidazole	172.0/128.0	172.0/128.0	25	15	3.69	2.70
	172.0/81.9			25		
Tinidazole	248.0/92.8	248.0/92.8	30	20	6.63	5.23
	248.0/128.0			15		
Ornidazole	220.0/127.9	220.0/127.9	30	15	6.45	6.47
	220.0/81.9			25		
Ofloxacin	361.9/261.0	361.9/261.0	35	25	5.15	6.72
	361.9/221.0			35		
Ciprofloxacin	331.9/314.0	331.9/314.0	40	20	3.17	6.93
	331.9/231.0			35		
Enrofloxacin	360.0/245.0	360.0/245.0	35	25	5.37	7.35
	360.0/203.0			36		

**Table 3:** Linear range, regression equation, and correlation coefficients.

Compound	Linear range (ng/mL)	Calibration equation			Correlation coefficients, *R* ^2^		
		Standard solutions	Acacia honey	Jujube honey	Standard solutions	Acacia honey	Jujube honey
Metronidazole	10–100	*y* = 149.667*x* + 228.847	*y* = 84.2987*x* + 17.3078	*y* = 36.6167*x* + 33.0998	0.998	0.997	0.996
Tinidazole	10–100	*y* = 69.3581*x* + 46.8099	*y* = 47.1843*x* − 59.0018	*y* = 33.5304*x* + 8.3281	0.997	0.997	0.993
Ornidazole	10–100	*y* = 86.7363*x* + 162.9	*y* = 57.8037*x* + 8.9570	*y* = 38.1225*x* + 13.5625	0.999	0.999	0.992
Ofloxacin	10–100	*y* = 159.216*x* − 487.671	*y* = 62.2512*x* + 56.6425	*y* = 51.2493*x* + 5.2237	0.994	0.990	0.993
Ciprofloxacin	10–100	*y* = 79.9202*x* − 207.609	*y* = 40.8103*x* + 4.2569	*y* = 33.0771*x* + 25.5369	0.996	0.999	0.996
Enrofloxacin	10–100	*y* = 88.4074*x* − 347.422	*y* = 69.1707*x* + 7.48835	*y* = 42.9161*x* + 12.0416	0.995	0.998	0.992

**Table 4:** LODs and LOQs for the target compounds.

Compound	Acacia honey		Jujube honey	
	LOD/(*μ*g/kg)	LOQ/(*μ*g/kg)	LOD/(*μ*g/kg)	LOQ/(*μ*g/kg)
Metronidazole	0.64	2.13	0.83	2.77
Tinidazole	1.25	4.17	1.51	5.03
Ornidazole	1.41	4.70	1.58	5.27
Ofloxacin	0.77	2.56	0.99	3.30
Ciprofloxacin	0.92	3.07	1.17	3.91
Enrofloxacin	1.13	3.76	1.24	4.13

**Table 5:** Recovery rates and RSDs of target compounds from honey samples using by LC-MS/MS.

Compound	Concentration level (*μ*g/kg)	Recovery% (RSD%)	
		Acacia honey	Jujube honey
Metronidazole	5	82.7 (0.044)	81.0 (0.058)
	25	85.7 (0.031)	100.7 (0.050)
	50	86.1 (0.020)	99.0 (0.024)

Tinidazole	5	115.6 (0.050)	85.60 (0.027)
	25	87.2 (0.031)	100.5 (0.051)
	50	82.6 (0.012)	82.5 (0.039)

Ornidazole	5	113.3 (0.044)	86.8 (0.017)
	25	93.2 (0.049)	101.8 (0.036)
	50	85.2 (0.043)	108.9 (0.007)

Ofloxacin	5	116.7 (0.046)	82.9 (0.057)
	25	88.6 (0.054)	94.8 (0.039)
	50	81.9 (0.019)	106.5 (0.023)

Ciprofloxacin	5	83.1 (0.027)	83.1 (0.027)
	25	96.8 (0.040)	96.8 (0.040)
	50	116.8 (0.050)	81.8 (0.033)

Enrofloxacin	5	109.9 (0.053)	86.5 (0.063)
	25	115.9 (0.044)	103.9 (0.062)
	50	112.0 (0.031)	115.9 (0.020)

The repeatability and intermediate precision results were expressed as relative standard deviation (RSD); number of determinations (*n*) = 3.

**Table 6:** Concentrations of antibiotics detected in acacia and jujube honey.

Number	Concentration of target compounds (*μ*g/kg) (*n* = 3)					
	Metronidazole	Tinidazole	Ornidazole	Ofloxacin	Ciprofloxacin	Enrofloxacin
(1)	ND	ND	ND	ND	ND	ND
(2)	ND	ND	ND	ND	ND	ND
(3)	66.95	ND	ND	ND	ND	ND
(4)	ND	ND	ND	ND	ND	ND
(5)	ND	ND	ND	ND	ND	ND
(6)	ND	ND	ND	ND	ND	ND
(7)	ND	ND	ND	ND	ND	ND
(8)	ND	ND	ND	ND	ND	ND
(9)	ND	ND	ND	ND	ND	ND
(10)	ND	ND	ND	ND	ND	ND
(11)	ND	ND	ND	ND	89.43	ND
(12)	ND	ND	ND	ND	ND	ND
(13)	ND	ND	ND	ND	ND	ND
(14)	ND	ND	ND	ND	ND	ND
(15)	ND	ND	ND	ND	ND	ND
(16)	ND	ND	ND	ND	ND	ND
(17)	ND	ND	ND	ND	ND	ND
(18)	ND	ND	ND	ND	ND	ND
(19)	ND	ND	ND	ND	ND	ND
(20)	ND	ND	ND	ND	ND	ND
(21)	ND	ND	ND	ND	ND	ND
(22)	ND	ND	ND	ND	ND	ND
(23)	ND	ND	ND	ND	ND	ND
(24)	ND	ND	ND	ND	ND	ND
(25)	ND	ND	ND	ND	ND	ND
(26)	ND	ND	ND	ND	ND	ND
(27)	ND	ND	ND	ND	ND	ND
(28)	5.87	ND	ND	ND	ND	ND
(29)	ND	ND	ND	ND	ND	ND
(30)	ND	ND	ND	ND	ND	ND
(31)	ND	ND	ND	ND	ND	ND
(32)	ND	ND	ND	ND	ND	ND
(33)	ND	ND	ND	ND	ND	ND
(34)	ND	ND	ND	ND	ND	ND
(35)	29.35	ND	ND	ND	52.91	ND
(36)	ND	ND	ND	ND	ND	ND
(37)	ND	ND	ND	ND	ND	ND
(38)	ND	ND	ND	ND	ND	ND
(39)	ND	ND	ND	ND	ND	ND
(40)	ND	ND	ND	ND	ND	ND
(41)	ND	ND	ND	ND	ND	ND
(42)	ND	ND	ND	ND	ND	ND
(43)	6.12	ND	ND	ND	ND	ND
(44)	ND	ND	ND	ND	ND	ND
(45)	ND	ND	ND	ND	ND	ND

ND: not detected; numbers (1)–(33): acacia honey; numbers (34)–(45): jujube honey.
